# The complete mitochondrial genome of *Pseudoxenodon stejnegeri* (Squamata: Colubridae: Pseudoxenodontinae) and its phylogeny

**DOI:** 10.1080/23802359.2020.1835584

**Published:** 2020-11-13

**Authors:** Liyue Zhang, Hui Li, Shengqin Wang, Shuran Li

**Affiliations:** a The First Affiliated Hospital, School of Medicine, Zhejiang University, Hangzhou, China; b College of Life and Environmental Science, Wenzhou University, Wenzhou, China

**Keywords:** *Pseudoxenodon stejnegeri*, Pseudoxenodontinae, mitogenome, phylogenetic relationship

## Abstract

The mitochondrial genome sequence of *Pseudoxenodon stejnegeri* (Squamata: Colubridae: Pseudoxenodontinae) from Taishun County, Zhejiang Province, China, which is 18,475 bp in length and contains 25 tRNAs (including extra two tRNA-Tyr genes and extra one tRNA-Met gene), two rRNAs, 13 protein-coding genes and two identical control regions. The overall AT content of the mitogenome is 59.6% (A = 32.6%, T = 27%, C = 27%, G = 13.4%). In BI and ML phylogenetic analyses, the monophyly of the family Colubridae was well supported and *P. stejnegeri* was a basal clade of Colubridae.

The taxonomic position of Genus *Pseudoxenodon* (Squamata: Colubridae: Pseudoxenodontinae) remains controversial. Species of *Pseudoxenodon* are widely distributed throughout southern and southeastern Asia. *Pseudoxenodon stejnegeri* is widely distributed in southern China (Zhang and Huang 2013). In this study, we sequenced the complete mitochondrial genome of *Pseudoxenodon stejnegeri* and combined with the existing mitochondrial genome sequence of Colubroidea to discuss the relationship of Genus *Pseudoxenodon*.

The sample of *P. stejnegeri* (ZJTS20180528) was collected from Taishun (N 119.68°, E 27.71°), Zhejiang Province, China. The sample was identified and stored at −20 °C in College of Life and Environmental Science, Wenzhou University, China. Total genomic DNA was extracted from muscle tissue using Ezup Column Animal Genomic DNA Purification Kit (Sangon Biotech Company, Shanghai, China) and stored in the laboratory. In this study, the complete mitochondrial genome was obtained according to the modified universal primers (Liao et al. 2020; Zong et al. 2020). Subsequently, the remaining gaps were sequenced by designing species-specific primers according to previously obtained sequences. All PCR products were sequenced in both directions by the Sangon Biotech Company (Shanghai, China). The mitochondrial genome was deposited in GenBank with an accession number MW018358.

The complete mitogenome of *P. stejnegeri* is a circular DNA molecule with a total length of 18,475 bp. It contains 25 tRNAs (including extra two tRNA-Tyr genes and extra one tRNA-Met gene), two rRNAs, 13 protein-coding genes and two identical control regions. Three tRNA-Tyr genes are identical but the extra one tRNA-Met is different. The overall AT content of the whole mitogenome is 59.6%. In the 13 protein-coding genes, *ND4* gene is used GTG as the start codon, while the remaining 12 protein-coding genes used ATN (N stands for A, T, C, G) as the start codon. Some protein-coding genes are used TAA as a stop codon. However, *ND1* and *COXI* are used AGG as a stop codon, *ND2, ND4* and *ND4L* used TAG as a stop codon, *ND3* and *ND6* used AGA as a stop codon, and *COXIII*, Cytb and *ND5* end with an incomplete stop codon (T–).

To construct a phylogenetic relationship of *P. stejnegeri*, 39 mitochondrial genomes of 33 Squamata species including three mitochondrial genomes as outgroups (*Bungarus fasciatus*, *Naja kaouthia*, *Ophiophagus hannah*) were downloaded from GenBank. Phylogenetic relationships were reconstructed using the Bayesian inference (BI) method implemented in MrBayes version 3.1.2 (Huelsenbeck and Ronquist 2001) and maximum likelihood (ML) in RAxML 8.2.0 (Stamatakis 2014) based on 13 protein-coding genes. To select conserved regions of the putative nucleotide sequences, each alignment was analyzed with Gblocks 0.91 b (Castresana 2000) using default settings. In BI and ML phylogenetic analyses (Figure 1), the monophyly of Colubridae was failed to support because the Family Dipsadidae was clustered into Family Colubridae. The monophyly of Genus *Elaphe* was failed to support because the clade of *Pantherophis slowinskii* and *Pituphis catenifer sayi* was clustered into Genus *Elaphe*, whereas the monophyly of Genera *Hebius*, *Lycodon*, *Ptyas*, *Sibynophis* and *Thermophis* was well support. *P. stejnegeri* was a sister clade of ((Dipsadidae + *Stichophanes ningshaanensis*)+((Sibynophiinae + Natricinae)+Colubrinae)).

**Figure 1.**
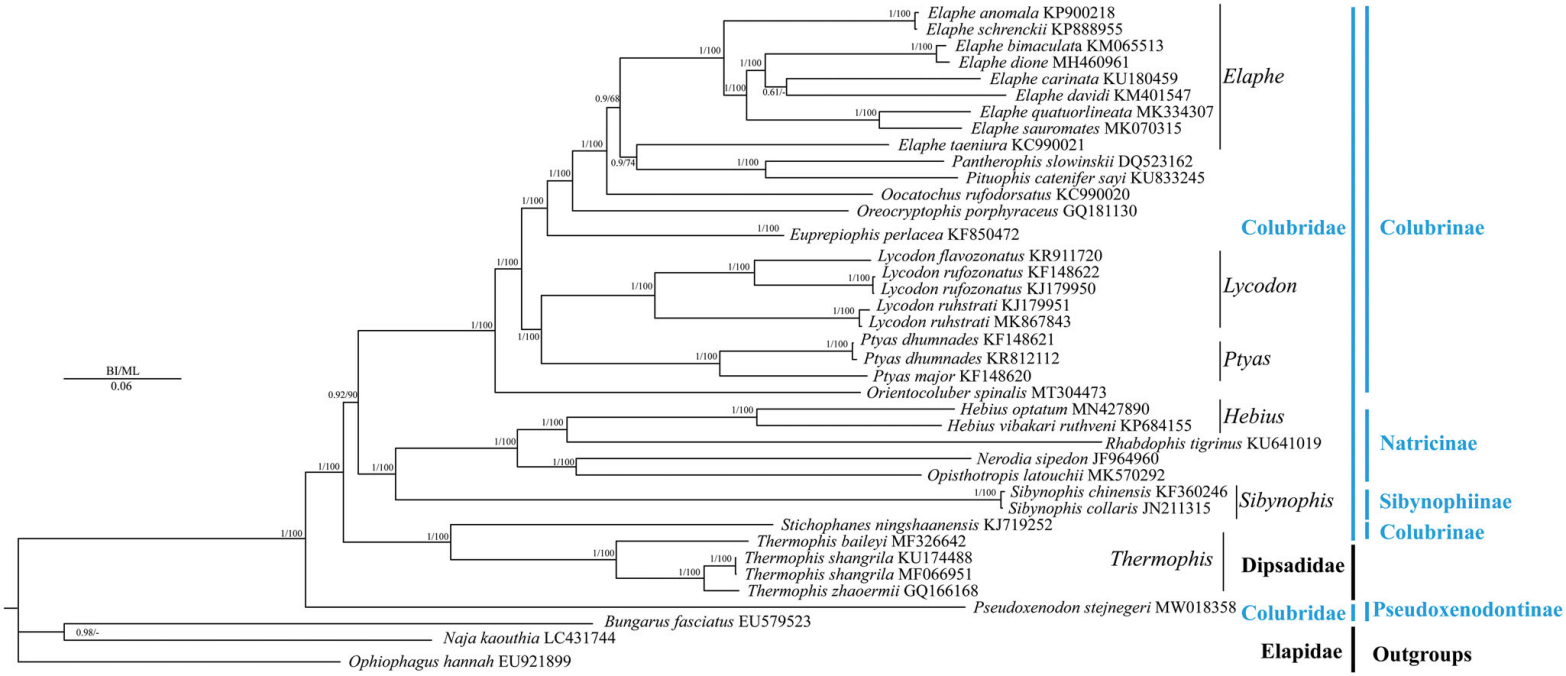
Phylogenetic tree of the relationships among 34 species of Colubridae including *Pseudoxenodon stejnegeri* in this study and three outgroups (*Bungarus fasciatus*, *Naja kaouthia*, *Ophiophagus Hannah*) (Singchat et al. 2019), were based on the nucleotide dataset of the 13 mitochondrial protein-coding genes. Numbers around the nodes are the posterior probabilities of BI (left) and the bootstrap values of ML (right). The GenBank numbers of all species are shown in the figure.

## Data Availability

The data that support the findings of this study are openly available in NCBI at https://www.ncbi.nlm.nih.gov/, reference number [MW018358].
